# A hybrid oxidation approach for converting high-strength urine ammonia into ammonium nitrate

**DOI:** 10.1016/j.wroa.2024.100277

**Published:** 2024-11-06

**Authors:** Zhiqiang Zuo, Tianyi Zhang, Xin Huang, Xiaotong Cen, Xi Lu, Tao Liu, Ho Kyong Shon, Min Zheng

**Affiliations:** a Water Research Centre, School of Civil and Environmental Engineering, University of New South Wales, Sydney, NSW 2052, Australia; b Australian Centre for Water and Environmental Biotechnology, The University of Queensland, St Lucia, QLD 4072, Australia; c Department of Engineering, King's College London, London WC2R 2LS, UK; d Department of Civil and Environmental Engineering, Graduate School of Engineering, Tohoku University, 6-6-06 Aramaki Aza Aoba, Aoba-ku, Sendai, Miyagi 9808579, Japan; e Department of Civil and Environmental Engineering, The Hong Kong Polytechnic University, Hong Kong 999077, China; f ARC Industrial Hub for Nutrients in a Circular Economy, Centre for Technology in Water and Wastewater, School of Civil and Environmental Engineering, University of Technology Sydney, NSW 2007, Australia

**Keywords:** Urine, Fertilizer, Partial nitritation, Chemical oxidation, Nutrient recovery, Circular economy

## Abstract

Nutrient resources contained in human urine have great potential to alleviate global agricultural fertilizer demand. Microbial nitrification is a recognized strategy for stabilizing urine ammonia into ammonium nitrate, a common fertilizer worldwide, but faces a core bottleneck of process instability due to microbial inhibition. This study reports a new approach by developing a hybrid oxidation process involving three stages—microbial ammonia oxidation, chemical nitrite oxidation and microbial nitrite oxidation. *Candidatus* Nitrosoglobus, a *γ*-proteobacterial ammonia oxidizer highly tolerant to free nitrous acid, was introduced in the first stage to oxidize half of the total ammonia in the influent (8 g NH_4_^+^-N/L) to nitrite. The nitrite was then chemically oxidized by using hydrogen peroxide via a rapid chemical reaction to form nitrate. The third stage, microbial nitrite oxidation, was employed to ensure the complete removal of residual nitrite following chemical oxidation. The overall concept demonstrated in this work showcased the robust performance of the hybrid system. Moreover, the system also had a dual advantage in achieving antimicrobial ability in the first and second stages, making treated urine a safe fertilizer.

## Graphical abstract

**Figure:**
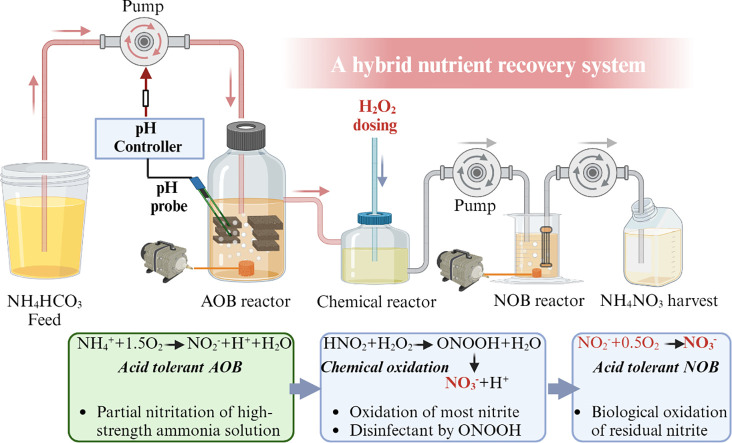

## 1 Introduction

Wastewater treatment plants (WWTPs) are experiencing substantial energy and chemical demands for nitrogen (N) and phosphorus (P) removal (Hao et al., 2015; Zhen et al., 2024). Compared with many upgrading approaches within WWTPs, a radical solution is to separate human urine from sewage, which can directly reduce 80 %–90 % of N and 50 % of P in mass loads of sewage (Maurer et al., 2006). Redirecting urine toward agricultural fertilization also holds immense promise with substantial environmental and economic benefits (Senecal et al. 2017; Martin et al., 2022; Wald et al., 2022). As such, urine diversion and conversion to fertilizers is a promising strategy to improve wastewater management and resource recovery (Hilton et al., 2020; Kavvada et al., 2017; Ishii et al., 2015). Although the direct use of urine as fertilizer has been extensive, it is not considered feasible in modern agriculture. Fresh human urine is characterized by a high urea concentration, approximately 7–9 g N/L, paired with a pH of approximately 6 (Udert et al., 2006). Upon collection, urea swiftly breaks down through urease activity, leading to the formation of ammonia and carbon dioxide (CO(NH_2_)_2_ + H_2_O→2NH_3_+CO_2_) (Udert et al., 2003a). This reaction escalates the urine pH to approximately 9, incurring significant gaseous ammonia emissions, generating an unpleasant smell and posing risks to human health (Ray et al., 2018; Udert et al., 2003a). Stabilization of urine nitrogen is thus crucial.

One stabilization approach is to inhibit urea hydrolysis and prevent the formation of ammonia, achieved by lowering or increasing the pH, but urea hydrolysis occurs too rapidly in urine collection systems (Udert et al., 2003a). The other approach allows urea hydrolysis to proceed while stabilizing ammonia to other nitrogen components through several technologies like ammonia stripping, adsorption, and ion exchange (Maurer et al., 2006). Microbial nitrification, involving microbial oxidation of ammonia to nitrite (NH_3_ + 1.5O_2_→NO_2_^−^ + *H*^+^ + H_2_O) by ammonia-oxidizing bacteria (AOB) and then to nitrate (NO_2_^−^ + 0.5O_2_→NO_3_^−^) by nitrite-oxidizing bacteria (NOB), has been proposed as a cost-effective strategy to stabilize urine ammonia (Christiaens et al., 2019; Sohn et al., 2024; Udert et al., 2003b). This process consumes urine alkalinity and lowers pH to around 6, eventually transforming volatile ammonia into a nonvolatile form of ammonium nitrate (NH_4_NO_3_), a widely used fertilizer (Udert et al., 2012). Additionally, other nutrients and elements such as K in urine can also be retained in the formed NH_4_NO_3_ liquid, enhancing its value as a fertilizer (Fumasoli et al., 2016; Udert et al., 2012; Zuo et al., 2023).

However, microbial nitrification of high-strength urine ammonia presents a significant challenge. Ammonia oxidation releases protons, which lowers the pH to around 6 (Udert et al., 2012; Zheng et al., 2017). This step often occasionally outpaces the second nitrite oxidation step, leading to nitrite accumulation. At low pH, a large share of nitrite is presented as free nitrous acid (FNA, NO_2_^−^+ *H*^+^↔HNO_2_). For instance, when treating urine with a high ammonia content of ∼8 g N/L, approximately 4 g N/L nitrite can form. This results in ∼10 mg HNO_2_/L of FNA at a pH of 6, which strongly inhibits AOB activity, with a 50 % reduction occurring at 0.42–1.72 mg HNO_2—_N/L (Zhou et al., 2011), ultimately resulting in the failure of microbial ammonia oxidation (Zheng et al., 2017; Zuo et al., 2022). Recent research on *γ*-proteobacterial ammonia oxidizers offers potential solutions to the FNA inhibition (Fumasoli et al., 2017; Zuo et al., 2024). Among these, *Candidatus* Nitrosoglobus, an AOB sourced from wastewater, has demonstrated remarkable resistance to FNA, with a 50 % activity inhibition concentration of 29.5 mg HNO₂-N/L, much higher than that of traditional AOB (Wang et al., 2021). This discovery suggests that *Ca.* Nitrosoglobus could be leveraged to enable efficient ammonia oxidation in environments with high FNA concentrations (Liu et al., 2024).

As a result of microbial ammonia oxidation, the subsequent conversion of the accumulated nitrite to nitrate poses another major challenge (Zuo et al., 2022, 2023). Microbial nitrite oxidation, mediated by NOB, is more strongly inhibited by FNA (Su et al., 2023), is highly unstable in systems with high nitrite concentrations (Meng et al., 2022; Zheng et al., 2018). In contrast, chemical oxidation of nitrite to nitrate is an alternative that can proceed efficiently at low pH levels (Udert et al., 2005), but this reaction becomes progressively slower as nitrite is consumed, resulting in incomplete conversion (Zuo et al., 2022). Therefore, a hybrid approach may be necessary, where chemical oxidation is employed to handle high nitrite concentrations, followed by microbial nitrite oxidation to achieve complete conversion of residual nitrite to nitrate.

**Fig. 1:**
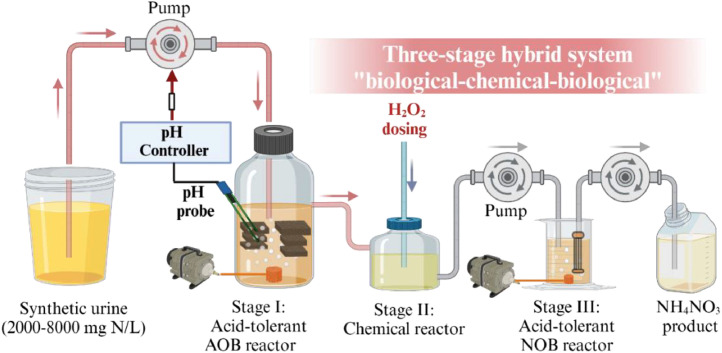
Diagram of a hybrid three-stage “biological–chemical–biological” urine treatment system.

This study aims to establish a novel hybrid three-stage “biological–chemical–biological” oxidation process for completely converting urine ammonia into NH_4_NO_3_ (Fig. 1). The first stage involves the introduction of *Ca.* Nitrosoglobus in a nitritation bioreactor (NH_4_^+^→NO_2_^−^). Subsequently, the produced nitrite is rapidly oxidized into nitrate (NO_2_^−^→NO_3_^−^) through chemical oxidation using hydrogen peroxide (H_2_O_2_). Finally, the residual nitrite remaining after chemical oxidation is completed converted to nitrate via microbial nitrite oxidation. The performance, stability, and efficiency of the hybrid approach were evaluated. The integrated system is expected to address a critical bottleneck in the conversion of urine ammonia into ammonium nitrate, presenting a stride toward a circular economy.

## 2 Results

### 2.1 Ammonium partial oxidation by *Ca*. Nitrosoglobus

**Fig. 2:**
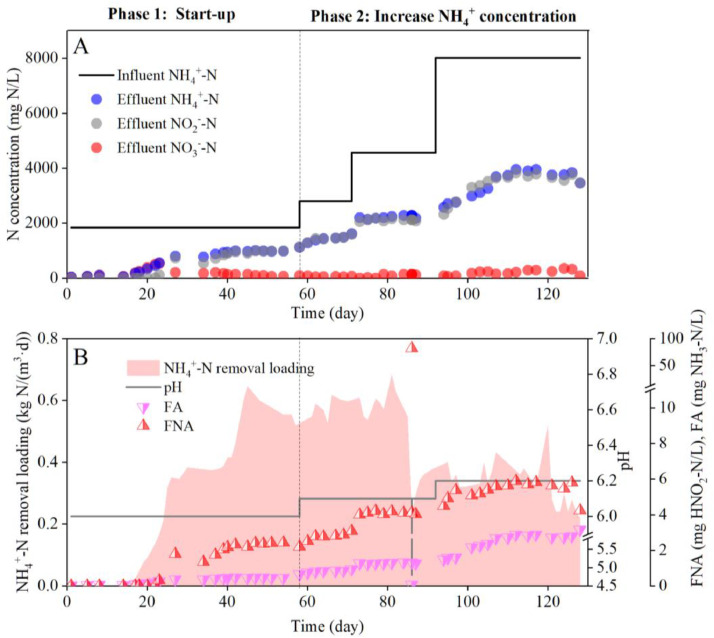
Nitrogen profiles (A), along with pH, FA, FNA and the NH_4_^+^-N/L removal loading rate (B) achieved in thed AOB bioreactor.

Fig. 2 shows the start-up and long-term maintenance of ammonium partial oxidation by *Ca.* Nitrosoglobus in the AOB bioreactor. During the initial two months of operation with an influent ammonium concentration of 2000 mg N/L, half of the ammonium was stably converted to nitrite. The ammonium and nitrite concentrations in the effluent both increased and stabilized at ∼1000 mg N/L on day 40 (Fig. 2A). The effluent nitrate concentration was <100 mg N/L. During days 61‒90, the influent ammonium concentration gradually increased to 2800, 4500, and 8000 mg N/L. The effluent ammonium and nitrite concentrations progressively increased and reached a level of 4000 mg N/L on day 110, after which they were sustained during the subsequent operational period.

However, on day 85, the pH sharply dropped to decreased to 4.5 due to an interruption of the influent, leading to an extremely high FNA concentration accumulated at ∼80 mg HNO_2—_N/L. This caused a sharp decrease in the ammonium removal loading rate. To reduce the inhibition effect of FNA on AOB, the FNA concentration was kept below 6 mg HNO_2—_N/L by controlling the reactor pH at 6.0‒6.2 (Fig. 2B). With this pH control strategy, the ammonium loading rate was stably maintained at a relatively stable level (0.34 ± 0.07 kg N/(m^3^·d)) even with a very high ammonium concentration of 8000 mg N/L in the urine wastewater. Together, these results demonstrate that applying *Ca.* Nitrosoglobus is a feasible approach for establishing stable performance of ammonia partial oxidation.

### 2.2 Hybrid oxidation of nitrite to nitrate

#### 2.2.1 Chemical oxidation of high-strength nitrite by H_2_O_2_

**Fig. 3:**
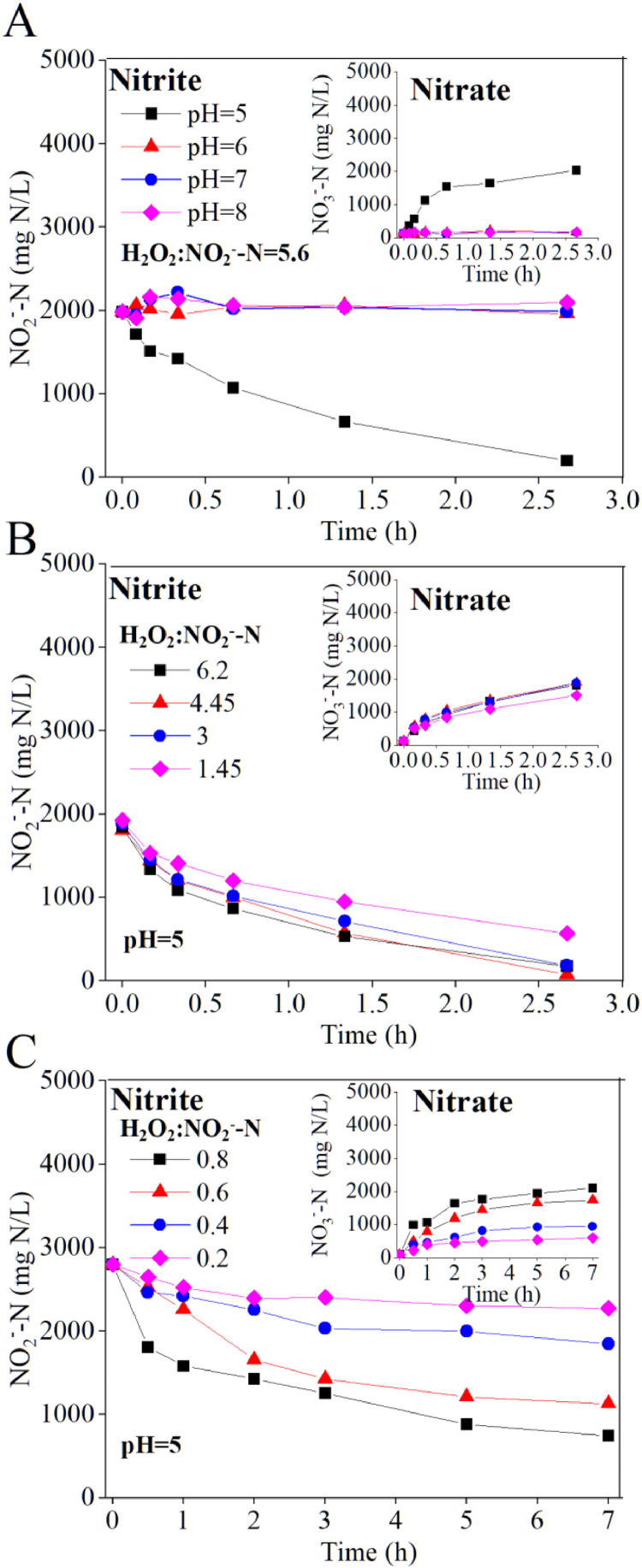
Chemical nitrite oxidation by H_2_O_2_ at different pH values (A) and H_2_O_2_ dosages with a set molar ratio of H_2_O_2_ to nitrite above 1 (B) and below 1 (C).

The second stage aimed at converting the effluent from the AOB bioreactor, which contained high-strength ammonium nitrite, into a solution of ammonium nitrate. Chemical nitrite oxidation using H_2_O_2_, a strong oxidant (Mavrikis et al., 2020; Shi et al., 2017, 2021; Viswanathan et al., 2015), was proposed to facilitate such conversion at a high rate. The chemical redox reaction between nitrite and H_2_O_2_ was first investigated using batch tests. Fig. 3A illustrates the results of nitrite oxidation at different pH levels (5‒8) and demonstrates that only pH 5 effectively activated the redox reaction. This may be because FNA serves as an actual reactant for the reaction with H_2_O_2_ (Vione et al., 2003), and more FNA forms at lower pH.

At pH 5, chemical nitrite oxidation was further tested with varying H_2_O_2_ concentrations. With an overdosing molar ratio of H_2_O_2_ to nitrite between 1.45 and 6.2, the average nitrite oxidation rate within 1 h was reached 2700‒3600 mg N/(L·h) (Fig. 3B). When the molar ratio of H_2_O_2_ to nitrite was below 1, the nitrite oxidation rate was dependent on the H_2_O_2_ dosage but varied within a low range (270‒980 mg N/(L·h)) (Fig. 3C). The molar amount of oxidized nitrite was almost equivalent to that of the produced nitrate (Fig. S3), indicating negligible intermediate nitrogen gas formation. This highlights a significant advantage of selecting H_2_O_2_ as an oxidant compared to earlier studies using O_2_ where harmful nitrogen oxide gas such as nitric oxide was produced (Udert et al., 2005). Additionally, ammonium concentration was unchanged in the batch tests (Fig. S4), leading to the formation of an NH_4_NO_3_ solution.

#### 2.2.2 Biological oxidation of residual nitrite

**Fig. 4:**
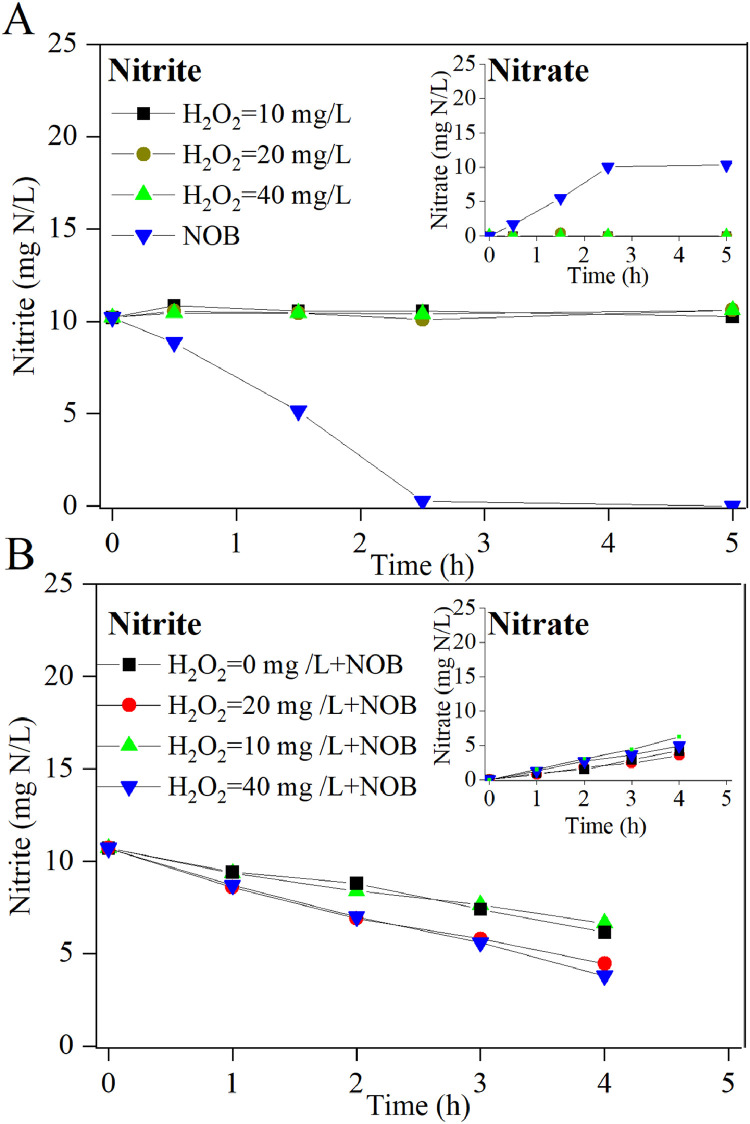
The oxidation of low-strength nitrite (10 mg N/L) by H_2_O_2_ and NOB (A) and the effect of H_2_O_2_ on NOB activity (B). The pH in all batch tests was 5.

As the nitrite concentration decreased, chemical oxidation slowed. When the initial nitrite concentration was 10 mg N/L, the nitrite oxidation reaction with different H_2_O_2_ dosages (10‒40 mg/L) became negligible at pH 5 (Fig. 4A). Since nitrite is toxic to plants and soil, any residual nitrite needs to be removed (van Staden et al., 1998). Therefore, the third stage was proposed to biologically oxidize residual nitrite to nitrate. The NOB bioreactor completely converted 10 mg N/L nitrite to nitrate in 2.5 h at pH 5 (Fig. 4A). A control aerobic experiment without NOB biomass conducted at pH 5 showed no nitrite consumption, suggesting that chemical nitrite oxidation with O_2_ could be negligible. These results demonstrated the feasibility of microbial nitrite oxidation as the final stage of the hybrid approach.

A concern was raised regarding the potential impact of residual H_2_O_2_ from the effluent of the second-stage chemical process on NOB activity in the third-stage microbial process. To verify this, a series of batch tests were performed at pH 5 to examine NOB activity in the presence of low H_2_O_2_ concentrations. The results indicated that introducing H_2_O_2_ at concentrations between 0‒40 mg/L did not suppress the NOB activity (Fig. 4B). Interestingly, H_2_O_2_ at 20 and 40 mg/L slightly enhanced NOB activity, although the potential mechanism needs further investigated.

### 2.3 Long-term performance of the integrated three-stage hybrid oxidation system

**Fig. 5:**
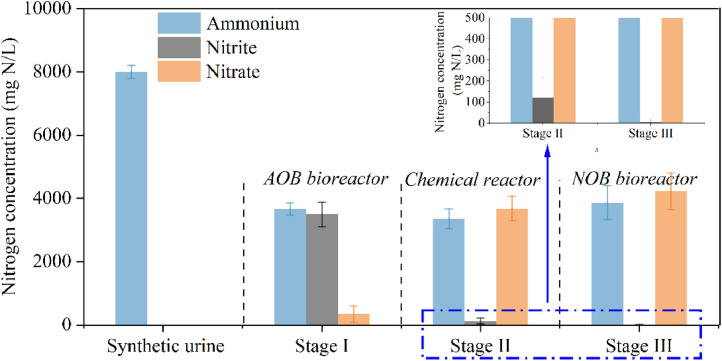
Long-term robust performance of the three-stage hybrid oxidation system. The small figure is a zooming-in version of the part in the blue dashed box to show the removal of residual nitrite more clearly.

The above results show that hybrid oxidation can effectively convert high-strength nitrite into nitrate. During days 130‒180 of operation of the AOB bioreactor, the three-stage hybrid approach was integrated. The long-term performance of the three reactors is presented in Fig. 5. In the first-stage FNA-tolerant AOB bioreactor, a 1:1 molar ratio of ammonium nitrite was produced. In the second-stage chemical reactor, ammonium nitrite was rapidly oxidized to ammonium nitrate within 4 h. The key conditions in the second-stage chemical reactor were a pH of 5 and a molar ratio of H_2_O_2_ to nitrite of 1:1, as determined through batch tests (Fig. 3). After chemical oxidation, the residual nitrite concentration was 120 ± 90 mg N/L. Nitrite was biologically oxidized in the third-stage NOB bioreactor. In the final treated urine, nitrite was absent, and the ammonium and nitrate concentrations were close. These results demonstrated the reliability of the hybrid oxidation approach for treating urine even with a very high concentration of ammonium (∼8000 mg N/L).

### 2.4 Antimicrobial potential

**Fig. 6:**
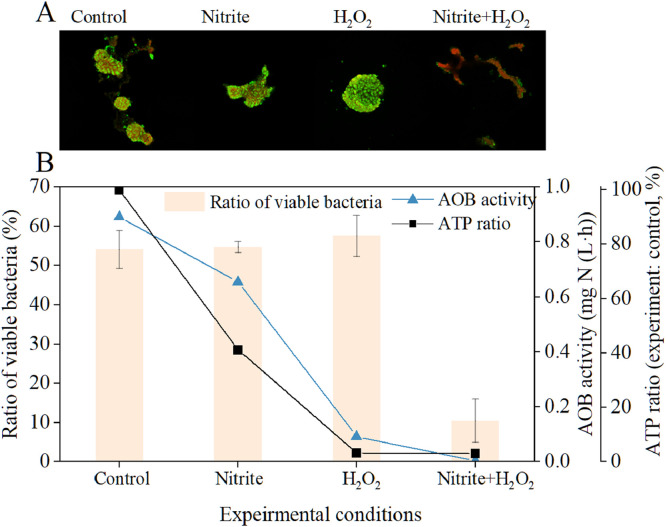
(A) Confocal laser scanning microscopy images of live/dead (green/red)-stained microbial cells under four different treatment conditions and (B) the live/dead ratio, ATP ratio and AOB activity.

An additional benefit of disinfection was expected in the hybrid oxidation approach due to the toxicity of nitrite/FNA and the strong oxidizing property of H_2_O_2_ (Duan et al., 2020; Jin et al., 2020). To assess disinfection efficiency, live/dead (green/red) staining of microbial cells, ATP analysis, and microbial activity measurements were conducted. Fig. 6A displays confocal laser scanning microscopy images of live/dead (green/red)-stained microbial cells. The result shows a red fluorescence image under the combined treatment of H_2_O_2_ and nitrite, indicating strong microbial inactivation. In contrast, the other three conditions still exhibited green fluorescence, a signal for live microbes. By quantifying green and red fluorescence intensities with ImageJ, the percentage of viable cells to total cells was calculated to be 50–60 % under three treatment conditions (control, only H_2_O_2_, only nitrite). The combined treatment of H_2_O_2_ and nitrite significantly decreased the percentage to 10 ± 5 % (Fig. 6B). Variations in ammonium oxidation and ATP activities are also shown in Fig. 6B. Under the combined treatment of H_2_O_2_ and nitrite, the ammonia oxidation activity was completely suppressed, and the ATP concentration decreased. These results, consistent with the live/dead results, collectively indicated the strong antimicrobial action of combining H_2_O_2_ and nitrite. This combination can form an intermediate product, peroxynitrous acid (ONOOH) (Vione et al., 2003), which acts as an antimicrobial agent against bacteria, fungi, and protozoa (Balazinski et al., 2021). Notably, H_2_O_2_ treatment alone caused a substantial decrease in AOB activity and ATP levels, although the change in the live/dead ratio was not remarkable. This suggests a likely "suppression" effect of H_2_O_2_ alone on microbes rather than "inactivation" at the tested H_2_O_2_ concentrations.

## 3 Discussion

**Fig. 7:**
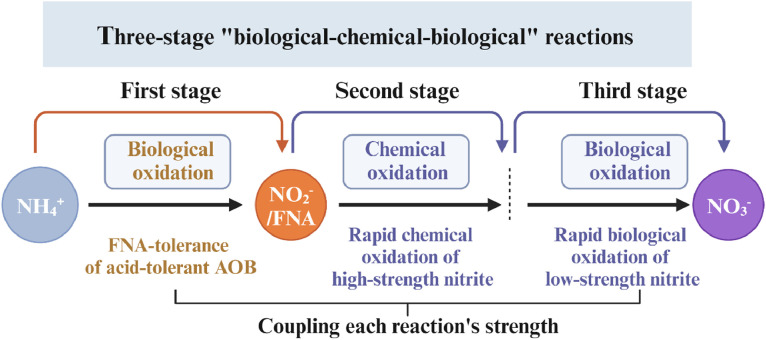
A novel solution entailed hybrid reactions for producing ammonium nitrate in urine.

This study develops a novel hybrid oxidation approach for stabilizing urine ammonia into NH_4_NO_3_ fertilizer. The first stage leverages the unique ability of *Ca.* Nitrosoglobus, a γ-proteobacterial ammonia oxidizer, to tolerate high levels of free nitrous acid (FNA), making it a promising solution for overcoming inhibition in ammonia oxidation. In the second stage, chemical oxidation using hydrogen peroxide (H₂O₂) successfully converts high concentrations of nitrite to nitrate, followed by a third stage where microbial nitrite oxidation removes residual nitrite. This approach builds on previous “biological-chemical” and “biological-biological” processes (Zuo et al., 2022,^2023^), which struggled with stability under high ammonia concentrations, by synergistically combining each stage's strengths to address specific challenges (Fig. 7).

This study demonstrates that chemical nitrite oxidation by H_2_O_2_ is a favorable option for nitrite oxidation to nitrate. In the field of atmospheric oxidative chemistry, redox interactions frequently occur between H_2_O_2_ and FNA to form HOONO (H_2_O_2_+HNO_2_→HOONO+H_2_O), which then rapidly decomposes and forms nitrate as the main transformation product (HOONO→NO_3_^−^+*H*^+^) (Vione et al., 2003). In this context, this study introduces HOONO to urine treatment and demonstrates that combining H_2_O_2_ and FNA is a feasible solution for nitrite oxidation. H_2_O_2_ solution is more attractive than O_2_ injection, as the latter causes significant nitrogen loss (Udert et al., 2005). In the reaction between H_2_O_2_ and FNA, the nitrite consumed fits the nitrate produced. However, further studies are needed to quantify the production of nitric oxide and nitrous oxide in this chemical reaction, as even small amounts could be critical.

A potential advantage of the produced fertilizer lies in its safe use. First, the presence of up to 6 mg N/L FNA in the AOB bioreactor can change the chemical structure and functional groups of organic molecules (Chislett et al., 2022). FNA-induced breakdown of macromolecular substances, pharmaceuticals, personal care products and antibiotic resistance genes can occur. For instance, FNA can effectively remove sulfamethoxazole by 95 % (Cheng et al., 2021). FNA also induces cell lysis (Wei et al., 2018; Xu et al., 2024), which may also support pathogen inactivation. Second, HOONO can form during chemical nitrite oxidation by H_2_O_2_. ONOOH can disinfect bulk liquids and biofilms against a variety of bacteria, fungi, and protozoa and can exert antimicrobial effects through nitration and oxidation of protein thiol cell components, DNA and membrane phospholipids and inhibition of the electron transport chain (Balazinski et al., 2021; Heaselgrave et al., 2010). As such, the chemical reactor may further reduce microbial risk in reusing treated urine. However, this chemical reaction may produce additional toxic by-products. For example, Jiang et al. (2013) reported that nitrogen dioxide (NO_2_) was an important toxic nitrogen intermediate generated in the mixture of FNA and H_2_O (Jiang et al., 2013). These aspects need more fundamental investigations in future research.

An economic analysis based on the experimental results was conducted to estimate the cost of the proposed approach (Text S3 and Table S1). Assuming urine collection from a building with 1000 people, each producing 1.5 L of urine (8 g N/L) per day, the annual NH_4_NO_3_ production is estimated at 4.4 tons of nitrogen, equivalent to 29.2 tons of NH_4_NO_3_ fertilizer (with an N content of 15 %). The total cost is estimated at $1120 per ton of NH_4_NO_3—_ N produced, which is lower than the price of commercial supply ($1466 per ton of NH_4_NO_3—_N). The cost of H_2_O_2_, which contributed to ∼80 % of the total cost, is a significant factor. It is possible to reduce H_2_O_2_ usage in the second stage by allowing higher nitrite concentrations to enter the third stage for microbial oxidation, potentially lowering overall costs. However, further research is needed to validate the stability and efficiency of microbial nitrite oxidation at these elevated nitrite levels. Recently, with H_2_O_2_ recognized as an important byproduct of water electrolysis in the emerging hydrogen industry (Mavrikis et al., 2020; Shi et al., 2017, 2021; Viswanathan et al., 2015), there may be opportunities to reduce chemical costs through system optimization across the water sector. Nonetheless, this economic analysis does not account for the costs of commercial-grade urine-diverting collection systems or NH_4_NO_3_ liquid concentration processes (e.g., distillation). Additionally, the produced fertilizer, containing organic material, may not be suitable for all conventional applications. A comprehensive economic and environment assessment, including larger-scale system optimization, is essential.

Lastly, this proof-of-concept study used synthetic urine, which is significantly less complex than real urine. Real urine contains over 2500 metabolites that differ greatly from that of synthetic urine (<15 metabolites) (Prithvi et al., 2024). Real urine contains a high organic component (about 10 g COD/L) (Udert et al., 2006), which could introduce additional challenges. These include longer HRT due to reduced oxygen availability for ammonia oxidation as well as potential membrane fouling (Kamranvand et al., 2018). Furthermore, non-biodegrade organic matter in the effluent of AOB reactor could affect the efficiency of the second-stage chemical nitrite oxidation, as H_2_O_2_ can chemically oxidize many types of non-biodegrade organic matter. Additionally, the buffer capacity of real urine differs greatly from that of synthetic urine, potentially resulting in different pH variations during the treatment process. Therefore, future studies must evaluate the feasibility of this approach in real urine systems to ensure its practicality and effectiveness.

## 4 Conclusion

This study demonstrates the reliability of a hybrid oxidation process for urine management. The microbial ammonia oxidation can leverage FNA-tolerant AOB to stably convert high-strength urine (∼8000 mg NH_4_^+^-N) into ammonium nitrite solution. Chemical nitrite oxidation can efficiently convert nitrite to nitrate and also enhance the antimicrobial properties of the resulting fertilizer. The microbial nitrite oxidation plays a role in ensuring a complete conversion of residual nitrite. The overall concept showcases robust performance of a hybrid urine treatment system, offering a promising stride toward a circular economy.

## 5 Materials and methods

### 5.1 Source of microorganisms

The nitrifying microorganisms utilized in the experiments involved AOB *Ca.* Nitrosoglobus and NOB Nitrobacter, which were enriched in our laboratory. In brief, *Ca.* Nitrosoglobus was cultivated using a membrane bioreactor (MBR) with a working volume of 10 L. The reactor pH was maintained at 5, and the average ammonia oxidation rate was 0.138 kg N/(m^3^·d) (Fig. S1). Nitrobacter was cultivated using another MBR with a working volume of 4 L and operated at a pH of 5. The influent nitrite concentration was set at 100 mg N/L, and effluent nitrite was not detected. The average nitrite oxidation rate was 0.058 kg N/(m^3^·d) (Fig. S2).

### 5.2 Setup and operation of the hybrid process

The hybrid urine treatment system comprised a AOB bioreactor, a chemical oxidation reactor, and a NOB bioreactor (Fig. 1), as detailed in the Supplementary Material (Text S1). Analytical methods are presented in Text S2.

### 5.3 Batch experiments to determine nitrite oxidation conditions

#### 5.3.1 Batch experiment I: chemical oxidation of nitrite by H_2_O_2_

A series of batch experiments were conducted to ascertain feasible conditions for chemical nitrite oxidation by H_2_O_2_. During days 61–90, the effluent of the AOB bioreactor was transferred to 150 mL conical flasks. In the first test, the impact of pH variation was investigated at different pH values of 5, 6, 7, and 8. The initial nitrite and H_2_O_2_ concentrations were maintained at 2000 mg N/L and 27,200 mg/L, respectively, with a molar ratio of H_2_O_2_ to nitrite of approximately 5.6. The second test aimed to explore the optimal H_2_O_2_ dosage. The initial molar ratio of H_2_O_2_ to nitrite was categorized into two groups: higher than 1 (four tests including 1.45, 3, 4.45, and 6) and lower than 1 (four tests including 0.2, 0.4, 0.6 and 0.8). H_2_O_2_ solution (35 % w/w) was used to obtain the above initial H_2_O_2_ concentrations. The initial nitrite concentration was maintained at 2000–3000 mg N/L.

#### 5.3.2 Batch experiment II: chemical and biological oxidation of nitrite

Batch tests were carried out to compare chemical and biological oxidation rates at low-strength nitrite concentrations. Three chemical tests were performed at initial H_2_O_2_ concentrations of 10 mg/L, 20 mg/L and 40 mg/L, with an additional test performed biologically with NOB inoculation. Each batch test took place in a 100 mL conical flask, and the same initial nitrite concentration of 10 mg N/L was used.

#### 5.3.3 Batch experiment III: potential effect of H_2_O_2_ on NOB activity

This set of batch tests was designed to investigate whether residual H_2_O_2_ from the chemical reactor harmed NOB activity. Four tests were conducted in 150 mL conical flasks with identical amounts of NOB biomass and the same initial nitrite concentration of 10 mg N/L. Initial H_2_O_2_ concentrations of 0 mg/L, 10 mg/L, 20 mg/L and 40 mg/L were used. In all the above batch experiments, the pH was 5.0 ± 0.2, and during the experiments, mixed liquid samples were regularly taken for the analysis of ammonium, nitrite, and nitrate concentrations.

### 5.4 Antimicrobial tests

To evaluate the antimicrobial ability caused by H_2_O_2_ addition in the chemical reactor, four batch tests were performed in 150 mL conical flasks using the biomass collected from the AOB bioreactor. The experimental groups included a control group, an H_2_O_2_ dosing group, a nitrite dosing group, and a combined dosing group. The concentrations were set at 4800 mg/L for H_2_O_2_ and 2000 mg N/L for nitrite (1:1 molar ratio), mimicking their typical levels in chemical reactors. The pH was set at 5.0 ± 0.2. After a two-hour treatment, 50 mg N/L ammonium was added to four flasks to measure ammonium oxidation rates (mg N/(L·h)). Biomass immediately collected in a 2 mL sterile tube was used for live/dead staining and adenosine triphosphate (ATP) analyses.

## CRediT authorship contribution statement

**Zhiqiang Zuo:** Writing – original draft, Visualization, Investigation, Formal analysis, Data curation, Conceptualization. **Tianyi Zhang:** Investigation, Data curation. **Xin Huang:** Visualization, Data curation. **Xiaotong Cen:** Investigation, Data curation. **Xi Lu:** Investigation, Data curation. **Tao Liu:** Writing – review & editing, Conceptualization. **Ho Kyong Shon:** Writing – review & editing, Conceptualization. **Min Zheng:** Writing – review & editing, Validation, Supervision, Funding acquisition, Conceptualization.

## Declaration of competing interest

The authors declare that they have no known competing financial interests or personal relationships that could have appeared to influence the work reported in this paper.

## Data Availability

Data will be made available on request

## Appendix Supplementary materials

Image, application 1
