# Supplementary material for: A hybrid oxidation approach for converting high-strength urine ammonia into ammonium nitrate
Source: Water Res X. 2024 Nov 6;25:100277. doi: 10.1016/j.wroa.2024.100277 (PMC11582551; doi:10.1016/j.wroa.2024.100277)
Supplement: Supplementary file 1 [file mmc1.docx]

**Supplementary Material**

**A hybrid oxidation approach for converting high-strength urine ammonia into ammonium nitrate**

Zhiqiang Zuo ^1,2,3^, Tianyi Zhang ^2,4^, Xin Huang ^2^, Xiaotong Cen ^2^, Xi Lu ^2^, Tao Liu ^2,5^, Ho Kyong Shon ^6^, Min Zheng ^1,2,*^

^1^ Water Research Centre, School of Civil and Environmental Engineering, University of New South Wales, Sydney, NSW 2052, Australia

^2^ Australian Centre for Water and Environmental Biotechnology, The University of Queensland, St Lucia, QLD 4072, Australia

^3^ Department of Engineering, King's College London, London WC2R 2LS, UK

^4^ Department of Civil and Environmental Engineering, Graduate School of Engineering, Tohoku University, 6-6-06 Aramaki Aza Aoba, Aoba-ku, Sendai, Miyagi 9808579, Japan

^5^ Department of Civil and Environmental Engineering, The Hong Kong Polytechnic University, Hong Kong 999077, China

^6^ ARC Industrial Hub for Nutrients in a Circular Economy, Centre for Technology in Water and Wastewater, School of Civil and Environmental Engineering, University of Technology Sydney, NSW 2007, Australia

***Corresponding author**: [min.zheng1@unsw.edu.au](mailto:min.zheng1@unsw.edu.au) (Min Zheng)

**Text S1. System setup and operation.**

*AOB bioreactor: ammonia partial oxidation to nitrite.* The AOB reactor was configured with a total liquid volume of 2 L and underwent the following steps. On day 0, 500 mL of *Ca.* Nitrosoglobus-enriched biomass was inoculated. Moreover, five sponge carriers, each measuring 2 cm × 2 cm ×1 cm, were added to facilitate the retention of the inoculated biomass. A synthetic feed was prepared using ammonium bicarbonate (NH_4_HCO_3_) to mimic urine wastewater with a 1:1 molar ratio of ammonium nitrogen and alkalinity. Starting with an initial concentration of 1800 mg N/L (Day 0-60), the influent ammonium nitrogen progressively increased to 2800, 4500, and 8000 mg N/L (Day 61-90) and stabilized at 8000 mg N/L (Day 91-180) to mimic the highest-strength nitrogen level in urine. A programmable logic controller (PLC) was used to regulate the *in situ* pH in the AOB bioreactor. When the pH decreases to 6, the feeding pump is intermittently activated, leveraging urine alkalinity buffer to increase the pH until the pH reaches the set point of 6.2. As a result, a pH range of 6.0-6.2 was achieved in the AOB bioreactor.

*Chemical oxidation reactor: high-rate chemical oxidation of nitrite to nitrate by H_2_O_2._* To oxidize nitrite generated from the AOB bioreactor, a chemical reactor with H_2_O_2_ dosing was designed during days 130-180. Key operating parameters, including pH and H_2_O_2_ dosage, were determined through independent batch tests (refer to batch experiments to determine nitrite oxidation conditions). Based on the batch test results, the pH in the chemical reactor was maintained at 5.0.

*NOB bioreactor: biological oxidation of residual nitrite to nitrate.* The NOB bioreactor featured a working volume of 200 mL and was prompted to completely oxidize residual nitrite in the effluent of the chemical reactor. The plants were inoculated with 200 mL of NOB-enriched biomass. Effluent from the chemical reactor was directed into the NOB bioreactor at a hydraulic retention time (HRT) of 1 day. The pH was unchanged at 5.0 ± 0.2. Batch test results were also compared for the oxidation rates of low-strength nitrite using H_2_O_2_ and NOB (refer to Batch Experiments to Determine Nitrite Oxidation Conditions section).

The ammonium, nitrite and nitrate concentrations in the influent and effluent of the three reactors were measured 2-4 times a week. Free nitrous acid (FNA) and free ammonia (FA) concentrations were calculated based on pH, total nitrite nitrogen (TNN), total ammonium nitrogen (TAN), and temperature according to the following equilibrium equations.

FNA (HNO_2_, mg N/L) = TNN (mg N/L)/(10^pH^ × e^-2300 / (273+temp (℃))^) (1)

FA (NH_3_, mg N/L) = TAN (mg N/L) × 10^pH^ / (e^6344 / (273+temp (℃))^ + 10^pH^) (2)

**Text S2. Analytical methods.**

The mixed liquor samples were filtered through 0.45 μm disposable sterile Millipore filters (Merck). The concentrations of ammonium, nitrite, and nitrate were measured by using a Lachat QuickChem8000 Flow Injection Analyzer (Lachat Instrument, Milwaukee, WI).

The viability of the bacterial cells was determined using a LIVE/DEAD BacLightTM bacterial viability kit (L7012, Molecular Probes). Two stain reagents were included in the commercial kit. One is the SYTO-9 green-fluorescent nucleic acid stain, which can be used to label live bacteria. The other stain is the red fluorescent nucleic acid stain (propidium iodide, PI), which penetrates only bacteria with damaged membranes. The stained samples were prepared by collecting the biomass from antimicrobial experiments (refer to the Antimicrobial Tests section). Then, the biomass suspension (1 mL for each set) was transferred into 2 mL plastic centrifuge tubes with 3 μL SYTO-9 and PI mixture (equal volume mixing). The tubes were incubated in the dark at room temperature for 15 min. Five microliters of the prepared solution were placed onto microscope slides and viewed using a confocal laser scanning microscope (Zeiss LSM 710 BiG) equipped with a Krypton–Argon laser (488 nm) and two He–Ne lasers (561 and 633 nm) at 400× magnification. The quantification of the emission (green) and emission (red) fluorescence intensities was conducted with ImageJ (National Institute of Health, USA). The ratio of green fluorescence to total fluorescence represents the percentage of viable cells to total cells.

ATP levels were determined by mixing 100 μL of the suspended biomass with 100 μL of BacTiter-Glo™ Reagent (G8230, Promega Corporation, USA) in 96-well plates (Nunc A/S, Thermo Fisher Scientific, Denmark). The mixture was cultured at room temperature for 5 min before the test. The relative light intensity of the sample was determined using a CLARIOstar Plus microplate reader (Beckman Coulter, USA) with a self-defined protocol (luminescence mode, room temperature, no shaking, and an integration time of 0.2 s per well with no filter). The ATP content of the reactor solution after autoclaving at 121 °C for 30 min served as the background, and all tests were performed in triplicate.

**Text S3. Economic analysis.**

Economic analysis includes both Capex and OpeX. The infrastructure inputs of the urine treatment unit (collection tank and three-stage reactor tank) included pipe, steel, concrete, and excavation. Electricity was used for mixing, aeration, and pumping. The infrastructure input data were obtained from Cashman et al. (2014); the energy input and cost were calculated using data from our previous studies (Zheng et al., 2017); and the electricity consumption data for the operation of the urine bioreactor tank were obtained from Zheng et al. (2017). The capital cost of the tanks was estimated using a unit price per volume of approximately $60/m^3^. Life cycle inventory data for chemicals, electricity, and construction materials were obtained from the GaBi database. The price of electricity was set at $0.12/kWh from the literature (Law et al., 2015). The price of ammonium nitrate (which contains 15% nitrogen) was assumed to be $220/ton (Alibaba website). The price of 35% H_2_O_2_ was assumed to be $260/ton (Alibaba website).


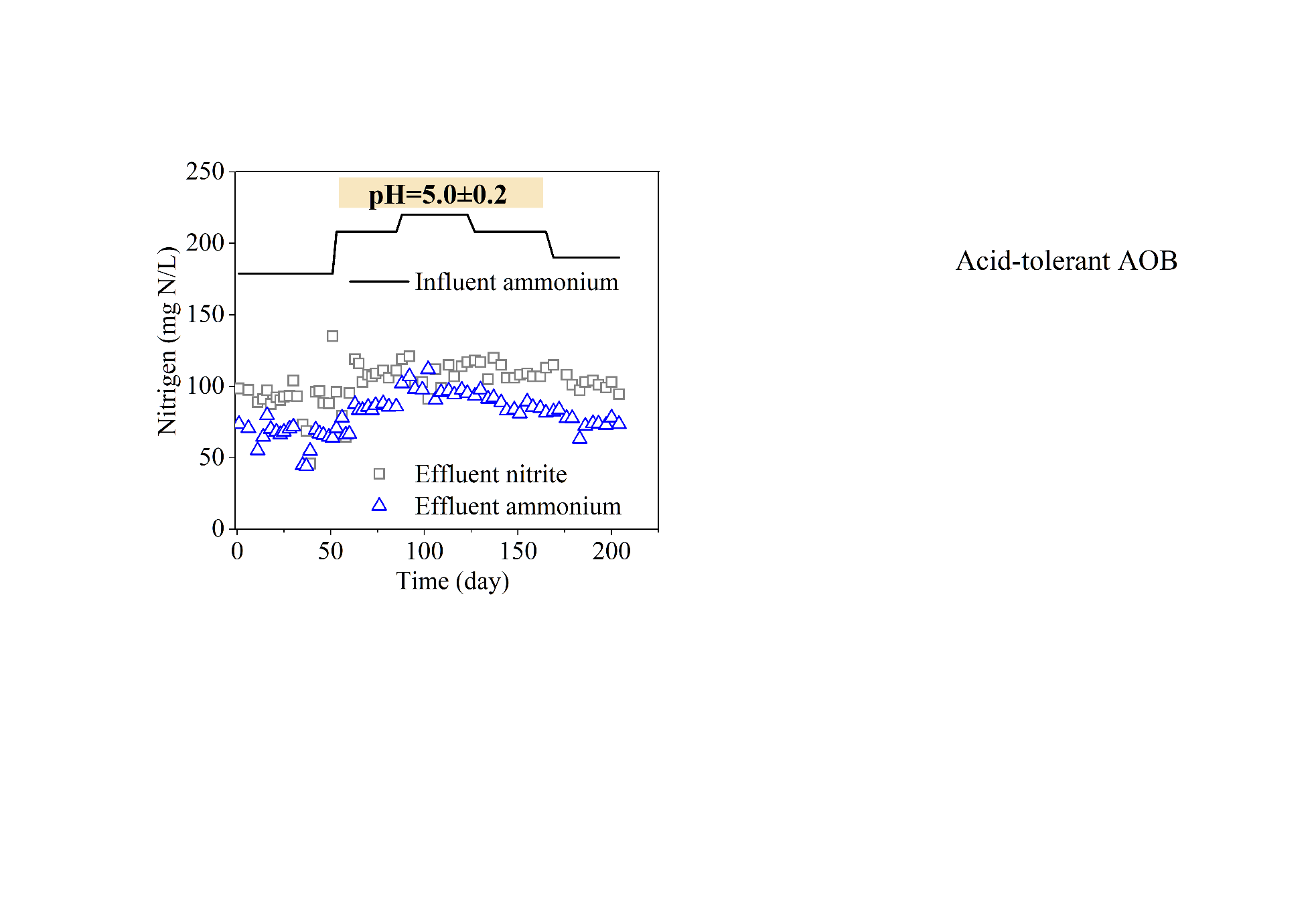


**Figure S1**. Nitrogen profiles in the AOB reactor operating at pH = 5.0 ± 0.2.


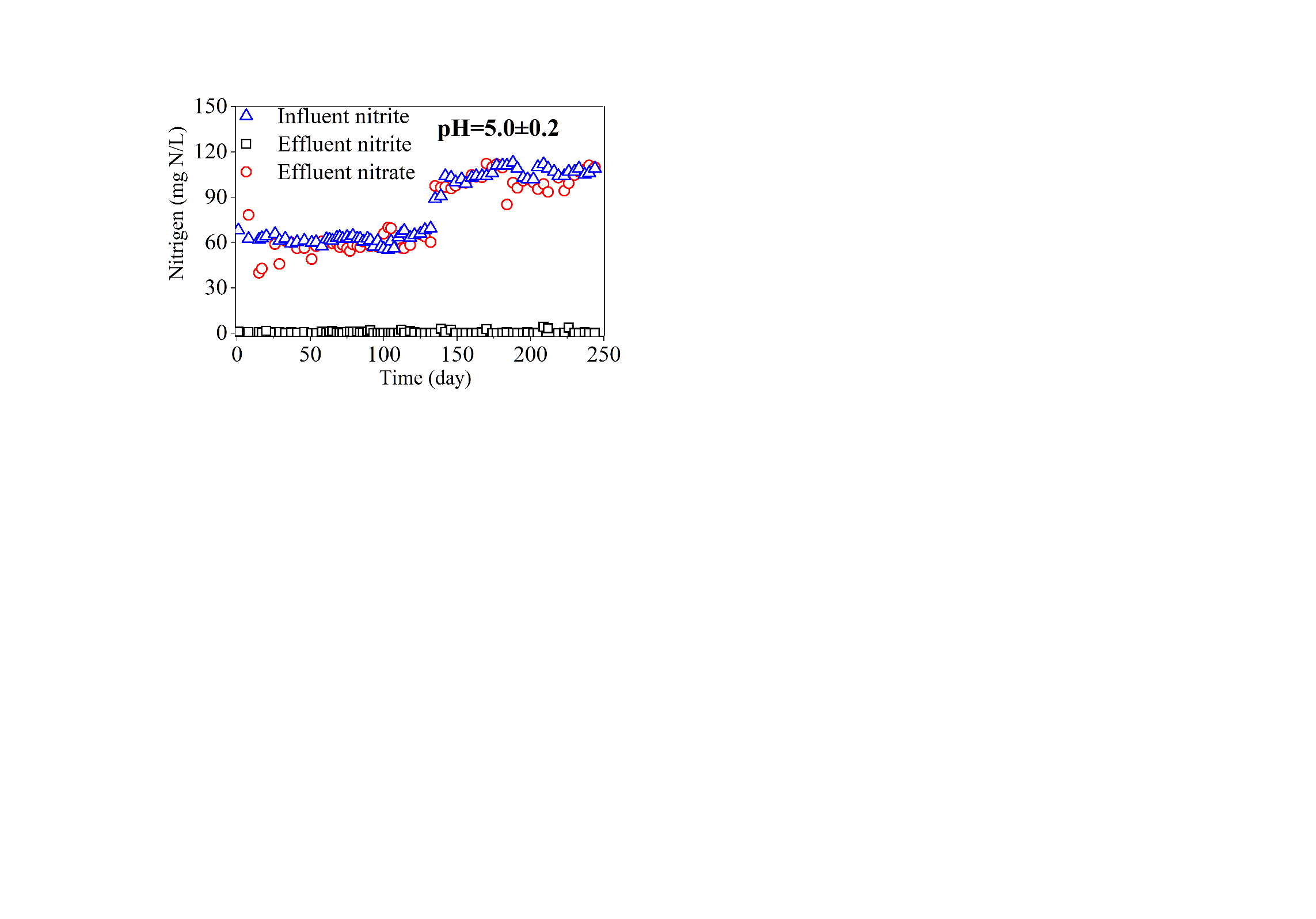


**Figure S2**. Nitrogen profiles in the NOB reactor operating at pH = 5.0 ± 0.2.


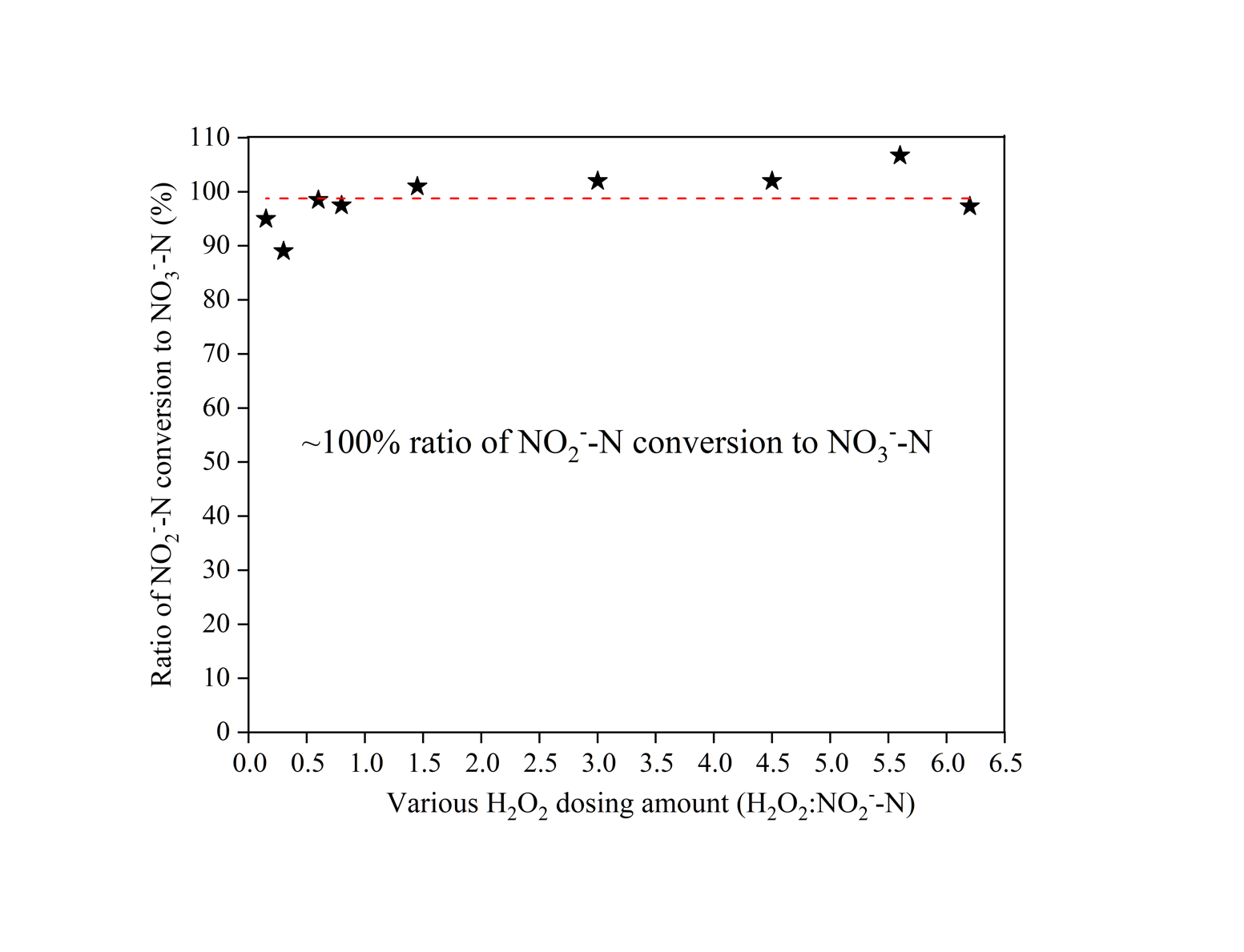


**Figure S3**. The ratio of NO_2_^-^-N conversion to NO_3_^-^-N based on chemical batch test results.


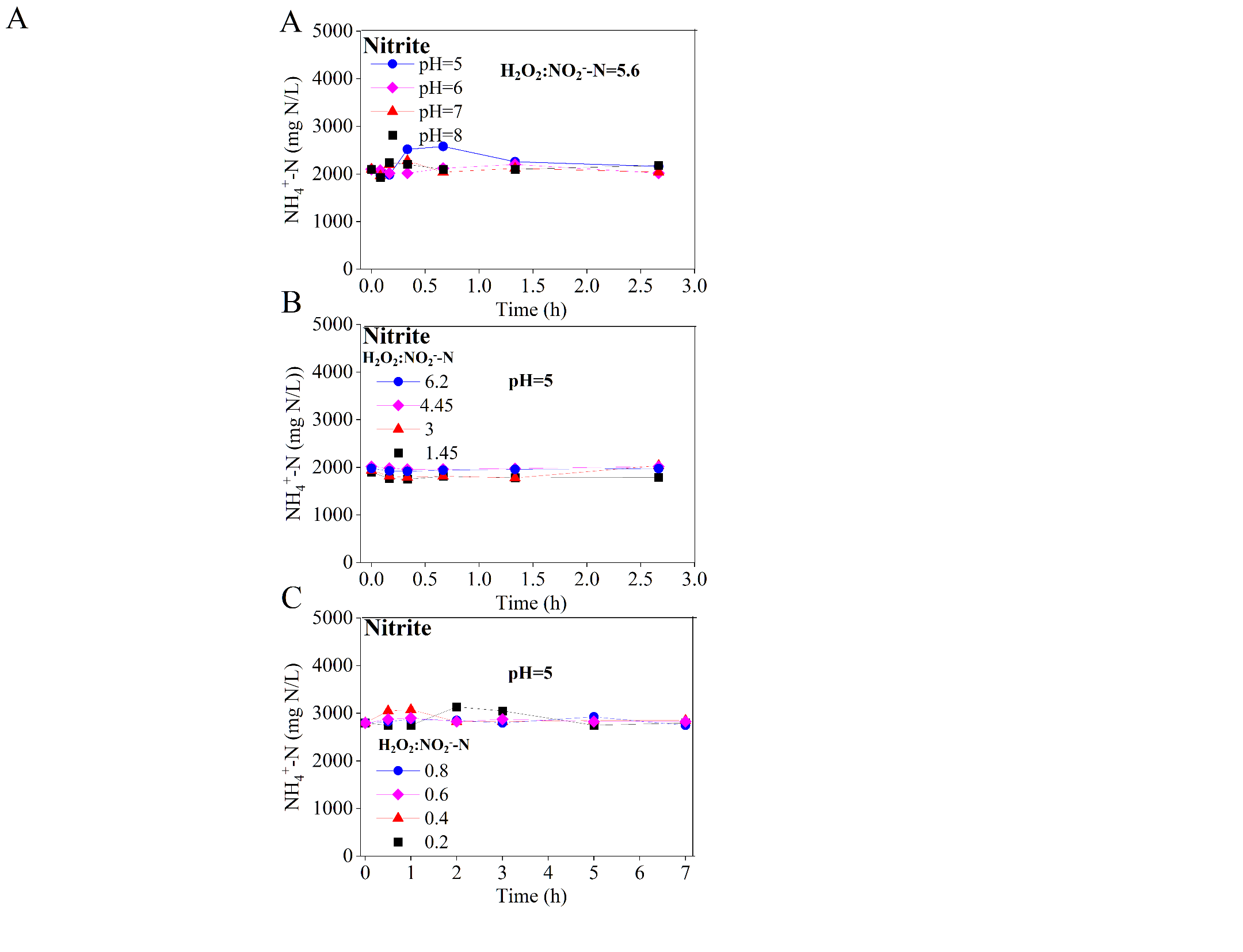


**Figure S4**. The variation of ammonium in the chemical nitrite oxidation by H_2_O_2_ at different pH values (A) and H_2_O_2_ dosages with a set molar ratio of H_2_O_2_ to nitrite above 1 (B) and below 1 (C).

**Table S1**. Economic analysis of the developed urine NH_4_NO_3_ recovery system

|  | **Parameter** | **Urine**  **NH_4_NO_3_** | **Commercial NH_4_NO_3_** |
| --- | --- | --- | --- |
| System assumptions | Lifetime (period over which capital costs are annualized) | 20 |  |
|  | Person population in the building | 1000 |  |
|  | Urine production volume per person (L/d/person) | 1.5 |  |
|  | Total urine production volume (m^3^/d) | 1.5 |  |
|  | Ammonium concentration in the urine (g N/L) | 8 g |  |
|  | Urine NH_4_NO_3_ production system (HRT and volume) | 14 d, 21 m^3^ |  |
|  | *AOB reactor tank (HRT and volume)* | 12 d, 18 m^3^ |  |
|  | *Chemical reactor tank (HRT and volume)* | 1d, 1.5 m^3^ |  |
|  | *NOB reactor tank (HRT and volume)* | 1 d, 1.5 m^3^ |  |
|  | **Total NH_4_NO_3_-N production (tonne N/year)** | **4.38** | **4.38** |
| Chemical input | H_2_O_2_ required for nitrite oxidation from AOB reactor | 1:1 |  |
|  | H_2_O_2_ required for per kg nitrite nitrogen (kg/NO_2_^-^-N) | 2.42 |  |
|  | H_2_O_2_ required for per kg total nitrogen (kg/total N) | 1.21 |  |
|  | **35% H_2_O_2_ dosing amount (tonne/year)** | **15.14** |  |
| Energy input | Energy for aerobic supply |  |  |
|  | O_2_/NH_4_^+^-N in AOB reactor (kg O_2_/kg N) | 3.43 |  |
|  | O_2_/COD in AOB reactor (kg O_2_/kg COD) | 1 |  |
|  | O_2_/ NO_2_^-^-N in NOB reactor (kg O_2_/kg N) | 1.14 |  |
|  | Electricity for aeration (kWh/kg O_2_) | 0.66 |  |
|  | Electricity for aeration (kWh/year) | 6,403 |  |
|  |  |  |  |
| Cost estimation | ① Capital cost of three tanks and major equipment ($) | 1,260 |  |
|  | Annualized capital cost ($/year) | 200 |  |
|  | ② Cost for electricity supply in reactor tank ($/year) | 768 |  |
|  | ③ Cost for H_2_O_2_ chemical supply ($/year) | 3,936 |  |
|  | ④ Cost for synthetic fertilizer market product ($/year) | 0 | 6,424 |
|  | **Total cost** (**$/year)** | **4,904** | 6,424 |
|  | **Total cost ($/tonne NH_4_NO_3_-N)** | **1,120** | **1,466** |

**Reference**

Cashman, S., Gaglione, A., Mosley, J., Weiss, L., Ashbolt, N.J., Cashdollar, J., Xue, X., Ma, C., Arden, S., 2014. Environmental and cost life cycle assessment of disinfection options for municipal wastewater treatment. EPA 600/R-14/377.

Law, Y., Ye, L., Wang, Q., Hu, S., Pijuan, M., Yuan, Z., 2015. Producing free nitrous acid – A green and renewable biocidal agent – From anaerobic digester liquor. Chemical Engineering Journal 259, 62–69.

Zheng, M., Zuo, Z., Zhang, Y., Cui, Y., Dong, Q., Liu, Y., Huang, X., Yuan, Z., 2017. Nitrite production from urine for sulfide control in sewers. Water Research 122, 447–454.
